# Backbone NMR resonance assignments for the VP1u N-terminal receptor-binding domain of the human parvovirus pathogen B19

**DOI:** 10.21203/rs.3.rs-4441481/v1

**Published:** 2024-06-04

**Authors:** Maria Luiza Caldas Nogueira, Renuk Lakshmanan, Gwladys Rivière, Mario Mietzsch, Antonette Bennett, Robert McKenna, Joanna R. Long

**Affiliations:** University of Florida; University of Florida; University of Florida; University of Florida; University of Florida; University of Florida; University of Florida

**Keywords:** Parvovirus B19, VP1u, receptor binding domain, NMR, PLA2, phospholipase A2

## Abstract

Parvovirus B19 (B19V) is a human pathogen that is the causative agent of several diseases in infants and adults. Due to a lack of antivirals against this virus, treatment options are limited. The minor capsid protein of B19V has a unique N terminus, named VP1u, which is essential for infection. The VP1u encodes a receptor binding domain (RBD), necessary for host cell entry, and a phospholipase A2 (PLA_2_) domain, crucial for endosomal escape during cellular trafficking. Both domains are indispensable for infection, making the RBD a plausible drug target for inhibitors against B19V, as it is located on the exterior surface of the virus. To date, no experimental structural information has been available for the VP1u component for any Parvovirus. Here we report the backbone NMR resonance assignments for the RBD of B19V and demonstrate it forms a stable structure. The backbone chemical shifts are in good agreement with a structure predicted by AlphaFold, validating that the RBD contains three helices connected by tight turns. This RBD construct can now be used for further NMR studies, including assignment of full-length VP1u, determination of protein-protein interaction interfaces, and development of B19 antivirals specific to the RBD domain.

**Database:** BMRB submission code: 52440

## Biological context

Human parvovirus B19 (B19V) is a linear single-stranded DNA virus that belongs to the genus *Erythroparvovirus* of the family *Parvoviridae*^1^. It is the causative agent of several human diseases, including hydrops in fetus, fifth disease in children, transient aplastic crisis in patients with sickle cell disease, and severe anemia in immune-compromised patients^2^. B19V packages a 5.6 Kb genome within a T = 1 icosahedral capsid^3,4^. The genome encodes two structural proteins, VP1 (86 KDa) and VP2 (60 KDa) which share the same C terminus. However, VP1, the minor capsid protein, has a unique N terminus referred to as VP1u extending 227 amino acids in length. VP1u comprises two domains, a receptor binding domain (RBD) and a phospholipase A2 (PLA_2_) domain, both of which are required for viral infectivity [5],[6],[7],[8]. The RBD facilitates viral entry into host cells by binding to tyrosine-protein kinase receptor UFO (AXL), while the PLA_2_ domain plays a crucial role in the endo-lysosomal escape of these viruses, ensuring successful translocation of the viral genome to the nucleus^7,9,10^. Additionally, VP1u is presented on the surface of the viral capsid, serving as a target for host immune responses^11^.

To date, no experimental structural information is available for the full length VP1u of any parvovirus due to its highly dynamic nature and low copy number within their capsids. VP1u conformational flexibility has hindered its crystallization for X-ray crystallography studies and its low molecular weight of 25 kDa is challenging for cryo-EM studies. The RBD of VP1u, currently characterized in primate Erythroparvoviruses, spans amino acid residues 5–68, with the conserved amino acids in the PLA_2_ domain spanning residues 123–227^5,8,12^. The amino acids between these two domains are thought to provide a flexible linker connecting the RBD and PLA_2_ domains^13^. Previous biophysical studies of B19V VP1u domains have shown that the RBD exhibits an α-helical fold, whereas the PLA_2_ domain exhibits a molten globule-like conformation^13^. Our initial efforts to assign backbone resonances for the entire B19V VP1u construct via nuclear magnetic resonance (NMR) were unsuccessful due to signal overlap likely from the unstructured PLA_2_ domain. Since the RBD has been previously shown by CD analysis to be well-structured, we designed a shorter construct to entirely encompass this domain (amino acids 1–90), essentially splitting the VP1u sequence in half as a first step in its structural characterization^13^. This construct proved to have sufficient structure and resolution to enable chemical shift assignments. Importantly, the RBD is sufficient for binding the recently identified AXL B19V receptor, making it an attractive target for drug development.

We report the backbone chemical shift assignments (^13^Cα, ^13^Cβ, ^13^CO, ^15^NH, ^1^HN, ^1^Hα, ^1^Hβ) for this newly engineered B19V RBD construct. We have successfully assigned 92% of the protein backbone amide resonances from amino acids 3–90, along with 97% of the backbone alpha carbons. Secondary structure predictions based on these chemical shifts are in strong agreement with a structure predicted by AlphaFold. This work sets the stage for NMR measurements aimed at examining the interaction of RBD with its putative receptor thereby identifying potential target regions within the RBD for inhibitor development. Additionally, our data provides a robust foundation for solving the structure of RBD and will facilitate monitoring of structural changes in response to environmental triggers such as pH or quaternary interactions with other biomolecules.

## Methods and experiments

### Protein expression and purification

The receptor-binding domain (amino acids 1–90) of the B19V minor capsid protein, derived from isolate J35 (NIH GenBank ID AY386330.1), was cloned into pET30a vector using restriction enzymes NdeI and XhoI (New England Biolabs, Ipswich, MA, USA). The cloning resulted in the addition of amino acid residues L and E after the 90th residue of RBD due to the XhoI site. Furthermore, this construct included a C-terminal hexa-histidine tag for purification purposes and was expressed in E. coli BL21 (DE3) cells (New England Biolabs, Ipswich, MA, USA). Initially, 2 L of lysogeny broth media containing kanamycin was inoculated with 1% of pre-culture. Cells were propagated at 37°C and 200 rpm until their OD_600_ reached a value of 0.6 when they were harvested by centrifugation and subsequently resuspended into 1 L of minimal media containing ^15^NH_4_Cl and ^13^C-glucose^14^. The culture was incubated at 37°C and 200 rpm for 30 min before induction of protein expression for 5h by the addition of 1 mM IPTG (Thermo Fisher Scientific, Waltham, MA). Cells were then harvested and resuspended in lysis buffer containing 25 mM Tris-HCl, 500 mM NaCl, 10 mM Imidazole, pH 8, and lysed using an LM10 microfluidizer at 18,000 psi. The cell debris was removed by centrifugation at 12,000 *x* g for 30 min at 4°C. The clarified supernatant was applied to 0.75 mL of Ni-NTA resin (G-Biosciences, St. Louis, MO, USA) and incubated at 4°C for 1h. Following this, the resin was washed with 20 column volumes of lysis buffer and RBD was eluted with 5 column volumes of elution buffer (25 mM Tris-HCl, 500 mM NaCl, 400 mM Imidazole, pH 8). The eluent was then subjected to a second round of purification on a HiLoad 16/600 Superdex 75 pg size exclusion column (GE Healthcare, Chicago, IL, USA), pre-equilibrated in storage buffer containing 20 mM sodium phosphate buffer, pH 6.5, 50 mM NaCl, 25 mM L-arginine, and 25 mM L-glutamic acid. Sample purity was evaluated via SDS-PAGE gel with Coomassie staining. The pure protein was then concentrated using an Amicon ultra-15 centrifugal unit with a 3 KDa cutoff (Merck Millipore, Burlington, MA, USA) and stored at −20°C. Deuterium oxide (10% v/v) and 1 mM trimethylsilyl propanoic acid (TSP) were added to lock and reference the magnetic field, respectively, for NMR experiments.

### Structure Prediction for the Receptor Binding Domain

**Figure 1:**
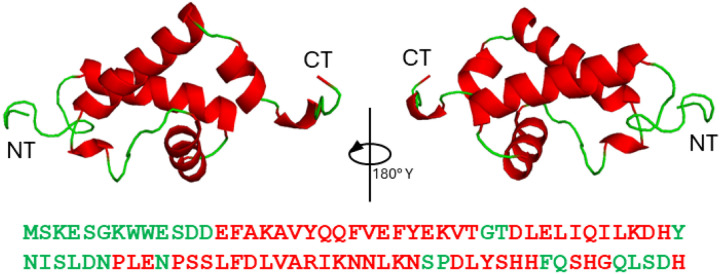
RBD structural model. The tertiary fold of RBD was predicated using AlphaFold. The model is colored based on its secondary structure with helices in red and coil regions in green. The right image is a 180° rotation of the left image.

To date, there is no experimentally determined structural data available for the RBD of B19V, therefore we used the AlphaFold2 structure prediction algorithm to model the RBD structure (Fig. 1). Computational predictions suggest that RBD is primarily α-helical with ~ 66% of its residues participating in α-helical secondary structural interactions. However, these α-helices are disrupted by several proline residues distributed in RBD`s primary amino acid sequence.

### NMR Data Collection

All NMR spectra were acquired at 298 K using an 800 MHz/54 mm bore Bruker Avance III spectrometer, equipped with a TCI cryo-probe. An initial ^1^H-^15^N HSQC spectrum demonstrated good dispersion of the amide resonances, indicative of a well-folded protein structure. The backbone assignments were facilitated by a suite of NMR experiments, including ^15^N-HSQC, ^13^C-HSQC, HNCO, HNcaCO, HNCA, HNcoCA, HNCACB, CBCAcoNH, and HBHAcoNH (Table 1). For chemical shift referencing, 1 mM trimethylsilyl propanoic acid was employed to directly reference proton chemical shifts and indirectly reference carbon and nitrogen chemical shifts. Data acquisition and processing were conducted using Topspin versions 3.6.5 and 4.3.0, respectively.

### Extent of Assignment and Data Deposition

**Figure 2:**
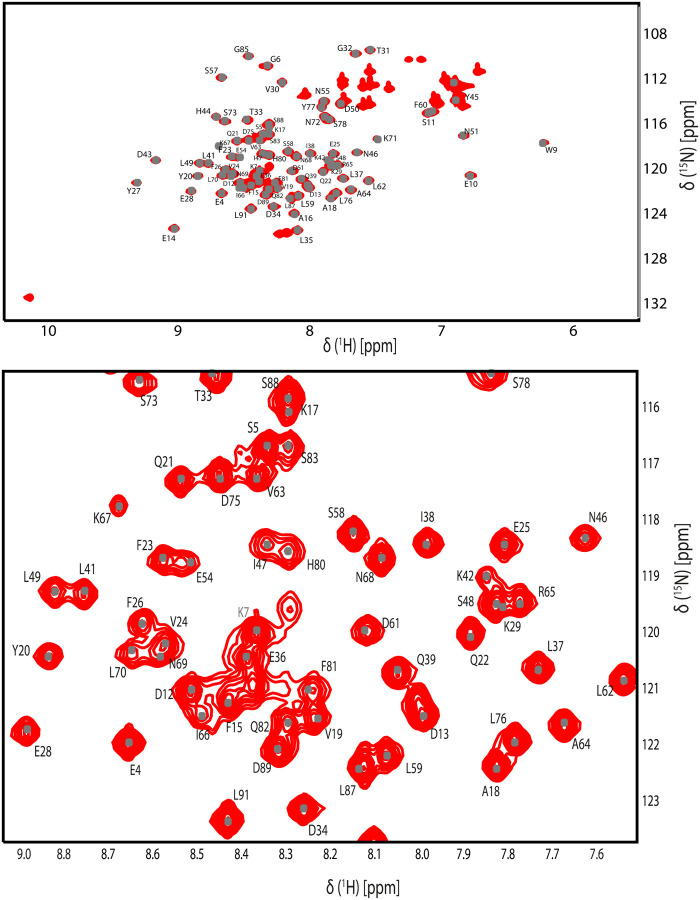
^1^H-^15^N-HSQC of VP1u RBD domain from Parvovirus B19. Residues are labeled with one-letter amino acid code and their primary sequence position. The upper panel shows the full spectrum, and the lower panel shows its central area enlarged for better visualization.

The backbone chemical shifts of RBD were determined using CCPNMR V3.2^15^. These assigned resonances were subsequently deposited in the Biological Magnetic Resonance Data Bank (BMRB) under the deposit number 52440. The ^1^H-^15^N HSQC spectrum shown in Fig. 2, indicates the backbone assignments. Within RBD amino acid sequence, three proline residues—P56, P74, and P52—were noted; however, these were not visible in the HSQC and only the CA, CB, and CO shifts for P56 and P74 could be assigned.

Similarly, other residues such as K3, W8, I40, L53, H79, and H84 lacked assignments in the HSQC but had their backbone carbon resonances assigned. Additionally, the first two residues at the amino terminus and the C-terminal His tag residues were not observed due to their dynamics. Overall, we successfully assigned ~ 99% of the backbone resonances from K3 to H90.

**Figure 3:**
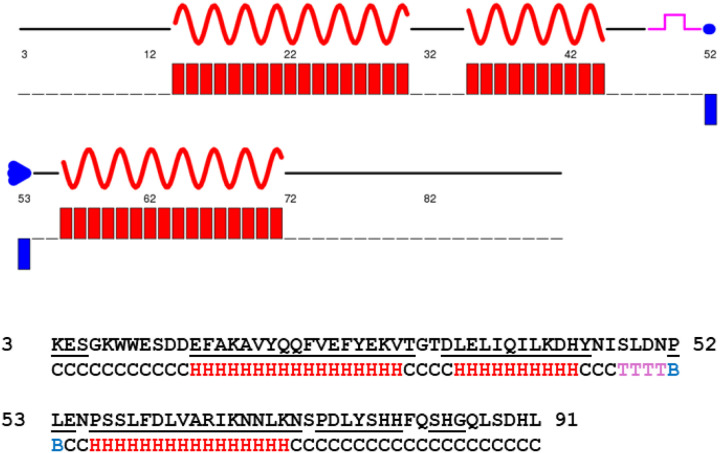
RBD secondary structure prediction based on assigned backbone chemical shifts. The secondary structure of RBD was calculated using CSI 3.0. Alpha-helix is shown in red, edge B-strand in blue, turn in magenta, and coil in black. The residues predicted to be helical by AlphaFold are underlined.

The chemical shifts assigned for the H, NH, CA, CB, CO, HA, and HB of the RBD domain were used as input to predict its secondary structure using Chemical Shift Indexing (CSI 3.0)^16^. This analysis revealed the presence of three α-helices within RBD (Fig. 3): H1 spanning E14 to V30, H2 from L35 to H44, and H3 from P56 to K71. Additionally, a turn was predicted between S48 to N51, with residues P52 to L53 predicted to be an edge β-strand, even though no β-sheet secondary structure was predicted for RBD by AlphaFold. Overall, these predictions are in good agreement with the AlphaFold structure, which indicated α-helices at positions E14-T31, D34-Y45, and P56-N72. AlphaFold also predicted short helical structures at K3-L5, P52-E54, P74-H80, S83-G85, and H90. However, the chemical shifts of these residues are more consistent with a random coil conformation (Fig. 3).

## Conclusions

We have shown that the heterologous expressed receptor-binding domain of the minor capsid protein VP1 from Human parvovirus B19 is well folded. Furthermore, we have successfully assigned the backbone chemical shifts, enabling the prediction of its secondary structure. These assignments will be used for future structure calculations, as well as a basis for future protein-ligand interaction studies.

## Data Availability

The Receptor-Binding Domain chemical shift values from Human parvovirus B19 were deposited in the Biological Magnetic Resonance Data Bank (BMRB) under accession code 52440.
